# Perioperative corticosteroids for reducing postoperative complications following esophagectomy: an updated systematic review and meta-analysis

**DOI:** 10.1186/s12893-024-02342-1

**Published:** 2024-02-15

**Authors:** Wan-wan Zou, Hsiao-Pei Mok, Qi-kun Zhu, Jing Luo, Song Yang, Jian-zheng Cen, Qiang Gao

**Affiliations:** 1 School of Medicine South China University of Technology, Guangzhou, 510006 People’s Republic of China; 2 Department of Cardiovascular Surgery, Guangdong Cardiovascular Institute, Guangdong Provincial People’s Hospital, Guangdong Academy of Medical Sciences, Southern Medical University, Guangzhou, 510080 People’s Republic of China; 3 Guangdong Provincial Key Laboratory of South China Structural Heart Disease, Guangzhou, 510080 People’s Republic of China; 4 Department of Breast Cancer, Cancer Center, Guangdong Provincial People’s Hospital, Guangdong Academy of Medical Sciences, Southern Medical University, Guangzhou, 510080 People’s Republic of China; 5 The Second School of Clinical Medicine, Southern Medical University, Guangzhou, 510515 People’s Republic of China

**Keywords:** Esophageal cancer, Corticosteroid, Esophagectomy, Efficacy, Safety

## Abstract

**Background:**

This updated systematic review and meta-analysis aims to evaluate the efficacy and safety of perioperative corticosteroid administration versus placebo for esophageal cancer patients following scheduled esophagectomy.

**Methods:**

We searched databases through June 30, 2023. We included articles on randomized controlled trials (RCTs) comparing perioperative corticosteroid administration with placebo in esophageal cancer patients with esophagectomy. The outcomes were the death rate during hospitalization, length of hospital stay, and short-term complications. Risk ratios (RRs) and corresponding 95% confidence interval (CIs) for each estimated effect size were applied for dichotomous outcomes, and the mean difference (MD) and corresponding 95% CIs for each estimated effect size were applied for continuous outcomes. We used GRADE to evaluate the quality of each of the outcome and the level of recommendations.

**Results:**

Nine RCTs with 508 participants were included in this study. Severe outcomes, including the length of hospital stay, leakage, mortality during the hospitalization period in the corticosteroid group was comparable to that in the control group, but positive effects of corticosteroid administration were observed on the length of intensive care unit stay (MD -3.1, 95% CI − 5.43 to − 0.77), cardiovascular disorders (RR 0.44, 95% CI 0.21–0.94) and other general complications (RR 0.49, 95% CI 0.29–0.85).

**Conclusions:**

Peri-operative intravenous corticosteroid administration may reduce cardiovascular disorders, other general complications and the length of ICU stay without carrying severe outcomes. More high quality RCTs are warranted to further investigate the effects of corticosteroids on postoperative mortality and complications for esophageal cancer patients with esophagectomy.

**Systematic review registration:**

Cochrane, registration number: 196.

**Supplementary Information:**

The online version contains supplementary material available at 10.1186/s12893-024-02342-1.

## Background

Esophagectomy is the established treatment for resectable esophageal malignancies at present. Unfortunately, although tremendous progress has been made in improving the outcomes with multiple approaches [1, 2], there is still a 5 to 10% of mortality rate and a 50% of morbidity rate associated with esophagectomy [3]. The complications-related costs also place a considerable burden to the family and society [4]. For esophageal carcinoma surgery, in general, the most common technical surgical complication is anastomotic leakage, and pneumonia is the most frequent non-surgical complication [5]. Nishiyama et al. reported that the infection complication rate could be as high as15.3% [6]. Furthermore, other complications such as hemorrhage, tracheobronchial leakage, chylothorax, reflux, malnutrition [7], tachyarrhythmias, and organ failure should also be taken into account. To reduce the incidence of postoperative complications, practitioners have adopted a large number of management strategies including pretreatment against specific risk factors [8, 9], intraoperative treatment [10–13], and postoperative symptomatic treatment [14–16].

The etiology of complications after esophagectomy varies. Senility, pulmonary disorders, poor cardiovascular conditions, malnutrition, and neoadjuvant therapy have been identified as risk factors [17]. The postoperative stress response after surgical procedures is also an important factor [18]. Oka et al implied that neutrophils might reflect the degree of inflammation and could be related to postoperative complications [19]. Kawamura subsequently investigated the relationship between inflammatory cytokines and postoperative complications, and found that an excessive systematic inflammatory response was likely to result in complications, especially organ failures [20]. There are many complications related to systemic inflammatory response syndrome [21, 22]. Therefore, reducing the degree of excessive systemic inflammatory response syndrome after surgery might be a significant challenge for practitioners. Indeed, strategies to attenuate systemic inflammation after esophagectomy have been a research focus for some time. Some previous studies focused on granulocyte colony-stimulating factor [23, 24]. Tanaka et al. investigated the role of synbiotics and found that they suppressed drastic inflammation probably through adjusting the intestinal microflora [25]. Ono et al. suggested that gabexate mesilate could be used to reduce the systematic inflammatory response [26]. Sivelestat was also reported to be an effective drug that improved the condition of patients with systemic inflammatory response syndrome [6, 27, 28]. A perioperative enteral diet supplemented with immune-enhancing substrates containing arginine, omega-3 fatty acids, and RNA was also proven to be a useful regimen [29]. Among the approaches used to minimize the stress response, perioperative corticosteroid therapy is the most beneficial for maintaining endocrine homeostasis. This is not only because corticosteroids are the standard anti-inflammatory and metabolic regulatory drugs, but also due to the large amount of research data available on this particular family of drugs [30, 31].

Many studies have investigated the role of perioperative corticosteroids in diminishing the stress response, and methylprednisolone is one of the most frequently used corticosteroids in the clinical settings [32–35]. Although it has been reported iteratively that perioperative corticosteroid therapy can inhibit inflammatory cytokine release and thereby improve prognosis [36–38], Yano et al. reported that the clinical benefits of preoperative steroid therapy were unclear [39]. Dexamethasone was reported to prolong coagulation time in rats [40]. Karwat et al. found a correlation between inhaled glucocorticosteroids and the prothrombin time [41]. Furthermore, glucocorticoids could induce immunosuppression, which is a potential risk factor for cancer relapse [42]. Due to these controversial conclusions, potential risks, lack of evidence regarding the benefits and harms, and concerns shared by many surgeons about delayed wound healing and potential cancer recurrence following the administration of corticosteroids in clinical settings, perioperative corticosteroid administration is currently not widely used. Although several meta-analysis reviews [30, 31, 43] have evaluated the effects of perioperative corticosteroid administration on esophageal cancer patients who underwent esophagectomy, the conclusion was still controversial. Furthermore, it is known that a systematic review is conducted to encompass all relevant studies on a specific issue. Therefore, it is imperative to update the literature of the systematic review after the emergence of new original research. Since the last meta-analysis, four more RCTs have been published, making it necessary to include them for reanalysis. In comparison to previous systematic review literature, this article has seen an increase in sample size, making the results more reliable. Therefore, an updated systemic review for clarification is necessary and important for this uncertain situation. This review aims to investigate the efficacy and safety of perioperative corticosteroid administration following esophagectomy.

## Methods

### Data sources and search strategies

Studies were identified by a literature search of MEDLINE (Ovid), the Cochrane Central Register of Controlled Trials (CENTRAL), the Cochrane Database of Systematic Reviews (CDSR), Embase (Ovid) and the Chinese Biomedical Literature Database (CBM) through June 30, 2023. The protocol and the search strategies have been published previously [44]. The publication languages were limited to English and Chinese. The protocol of this review has been registered in Cochrane with the registration number of 196.

### Eligibility and exclusion criteria for study selection

Only randomized controlled trials (RCTs) were included in this study. Eligible studies compared perioperative corticosteroid administration with placebo (isometric normal saline solution/no treatment) in esophageal cancer patients with esophagectomy. Studies with two or more groups of corticosteroids were also included as long as they included a no treatment/placebo control group. The types of corticosteroids included cortisone, hydrocortisone, prednisone, prednisolone, methylprednisolone, betamethasone, dexamethasone, and mineralocorticoid. Corticosteroids were administered during the hospitalization period, and the treatment duration could not exceed 10 days. If there was a pause in therapy, the duration could not exceed 5 days. The administration route could be oral or intravenous infusion.

The administration dosage equivalences were established according to the study by Haynes [45], i.e. prednisone 5 mg = prednisolone 5 mg = hydrocortisone 20 mg = cortisone 25 mg = triamcinolone 4 mg = methylprednisolone 4 mg = betamethasone 0.75 mg = dexamethasone 0.75 mg. We regarded methylprednisolone as the standard corticosteroid in this review and the doses of other corticosteroids were converted to a methylprednisolone-equivalent dosage.

### Data collection

Three of our review authors (WWZ, MHP, QKZ) scanned the title or abstract of every retrieved article independently. Any disagreements concerning study eligibility were resolved by consulting a third review author (GQ). We evaluated the authenticity of the randomization procedure by contacting the original study authors if necessary. Three authors (JL, SY) independently extracted data from eligible studies using a pre-standardized data extraction form. Any disagreements were resolved by consensus. If the data of any study were deemed insufficient, the corresponding authors were contacted for further information.

Primary outcomes were mortality during the hospitalization, length of hospital stay (defined as the time from operation to discharge), and length of hospital stay in the intensive care unit (ICU) due to an ill-defined consensus regarding postoperative complications [46]. Secondary outcomes were short-term complications including pulmonary disorders e.g. pneumonia, atelectasis, pleural effusion, pyothorax, and respiratory failure; cardiovascular disorders e.g. heart failure, arrhythmia including sinus tachycardia, extra systole, atrial fibrillation, and atrial flutter; anastomotic leakage, defined as the extravasation of water-soluble contrast medium and/or the appearance of oral ingested methylene blue in the thoracic drainage [13]; general infection complications e.g. systemic inflammatory response syndrome, wound infection, mediastinitis, sepsis, and urinary tract infection; renal failure; hepatic failure; other general complications (e.g. MOF, DIC, System multi organ failure in the ICU, Any organ system failure, the postoperative intubation period, bleeding, chylothorax etc.).

### Assessment of risk of bias and risk of publication biases

Three authors (JZC, WWZ, MHP) independently assessed the risk of bias for each study according to the criteria in the *Cochrane Handbook for Systematic Reviews of Interventions* including selection bias, performance bias, detection bias, attrition bias, reporting bias, and others [47]. Any disagreements were resolved by consultation.

We also used GRADE software to evaluate the quality of each of the outcome and the level of recommendations.

### Statistical analysis

For dichotomous data, the number of participants and the incidence of events in each group were extracted to calculate the relative risks (RRs) and their corresponding 95% confidence intervals (CIs). For continuous data, we calculated the mean differences (MDs) with their corresponding 95% CIs. Heterogeneity was assessed using the Chi^2^ test, with the statistical significance set at *P* < 0.1. The heterogeneity was assessed using the I^2^ statistic [47]. If substantial heterogeneity (I^2^ statistic > 40%) was found, sensitivity analyses were performed to explore the cause of the heterogeneity.

Statistical analysis was performed using Review Manager 5 with the random-effect model for anticipated substantial heterogeneity across studies regarding the mortality during hospitalization, pulmonary and cardiovascular disorders, anastomotic leakage, and general infection complications. We pooled the results as risk ratios (RRs) and calculated 95% CIs for each estimated effect size using the Mantel-Haenszel (MH) for dichotomous outcomes. Results in the form of MDs and the corresponding 95% CIs for each estimated effect size were applied for continuous outcomes.

## Results

### Search results and study characteristics

**Fig. 1:**
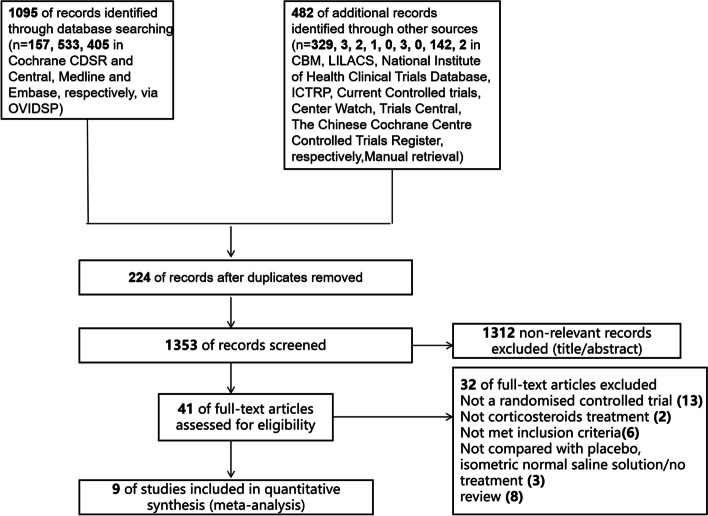
Study flow diagram

As shown in Fig. 1, after an exhaustive literature search of the aforementioned database resources,1577 articles were obtained. A total of 1312 articles were excluded for not meeting the review theme and inclusion criteria. The 41 remaining articles were further examined via full text reading by three authors (MHP, WWZ, GQ). Nine of the articles were found to fulfill the inclusion criteria for this review [32, 34, 35, 37, 39, 48–51].

Nine studies with a total of 508 participants were included. All of the studies were single-centered and had a two-arm parallel group design. Only the study by Afghani et al. was registered in the Iranian Registry of Clinical Trials and the protocol information was accessible [48].

As shown in Table 1, the sample sizes of included studies ranged between 17 and 128. Included studies were from three different countries: five studies from Japan [32, 34, 35, 37, 39], one study from Iran [48], and three studies from China [49–51]. The included studies varied regarding corticosteroid doses and administration time points. Methylprednisolone or methylprednisolone equivalent (prednisone in Gao et al. [49]) intravenous administration with pulse therapy dosage (> 200 mg/day) was applied before surgery in six studies [32, 34, 35, 37, 39, 49]. Afghani et al. applied intravenous methylprednisolone administration with a very high dose (125 mg/day) during the operation, specifically, at the end of esophagogastric anastomosis [48]. Corticosteroid administration was only performed once.

**Table 1:** Characteristics of included studies

First author (year)	Country	No. of Patients (M/F)	Age (years)	Intervention group (n)	Control group (n)	Treatment time
Afghani (2018)	Iran	60 (26/34)	59.85 ± 8.12	Methylprednisolone (125 mg, *n* = 30)	Blank control (n = 30)	At the end of esophagogastric anastomosis
Gao (2017)	China	90 (47/43)	Intervention group: 59.3 ± 3.2 Control group: 59.6 ± 3.6	Prednisone (10 mg/kg, n = 45)	Blank control (*n* = 45)	In the morning of surgery day
Matsutani (1998)	Japan	33^a^ (24/4)	64 ± 7	Methylprednisolone (10 mg/kg, *n* = 14)	Saline (*n* = 19)	At the time of anesthesia induction
Sato (2002)	Japan	66 (60/6)	Intervention group: 62 ± 8 Control group: 64 ± 7	Methylprednisolone (10 mg/kg, *n* = 33)	Saline (n = 33)	30 minutes before surgery
Takeda (1997)	Japan	30 (27/3)	Intervention group: 63 ± 7 Control group: 63 ± 10	Methylprednisolone (10 mg/kg, *n* = 15)	Saline (n = 15)	Before anesthesia induction
Takeda (2003)	Japan	17 (15/2)	Intervention group: 65 ± 1 Control group: 60 ± 3	Methylprednisolone (10 mg/kg, *n* = 7)	Saline (*n* = 10)	Before anesthesia induction
Yano (2005)	Japan	40 (36/4)	Intervention group: 64 ± 6 Control group: 56 ± 7	Methylprednisolone (500 mg, *n* = 20)	Saline (n = 20)	Within 2 h before surgery
Xu (2021)	China	128 (69/59)	Intervention group: 62.36 ± 3.04 Control group: 62.85 ± 3.15	Methylprednisolone sodium succinate (80 mg, *n* = 64)	Blank control (*n* = 64)	within 24 hours after surgery
Cao (2021)	China	44 (39/5)	Intervention group: 62.86 ± 5.22 Control group: 63.00 ± 6.19	Methylprednisolone (500 mg, *n* = 22)	Saline (n = 22)	before anesthesia induction

^a^The article did not identify the gender of the remaining five person

Six of the included studies [32, 34, 35, 37, 39, 48] reported mortality during the hospital stay but only one study [34] reported the length of hospital and ICU stay. Four studies [32, 34, 35, 48] ended before discharge. The follow up in the study by Sato [37] and Yano [39] lasted for 4 years and 5.5 years, respectively, but only short-term outcomes were extracted for meta-analysis. Gao [49] reported that the follow up period was 3 months.

Postoperative general infection complications were reported in all included studies. Other postoperative complications, such as pulmonary disorders, renal failure, etc. were not reported in all the studies.

**Fig. 2:**
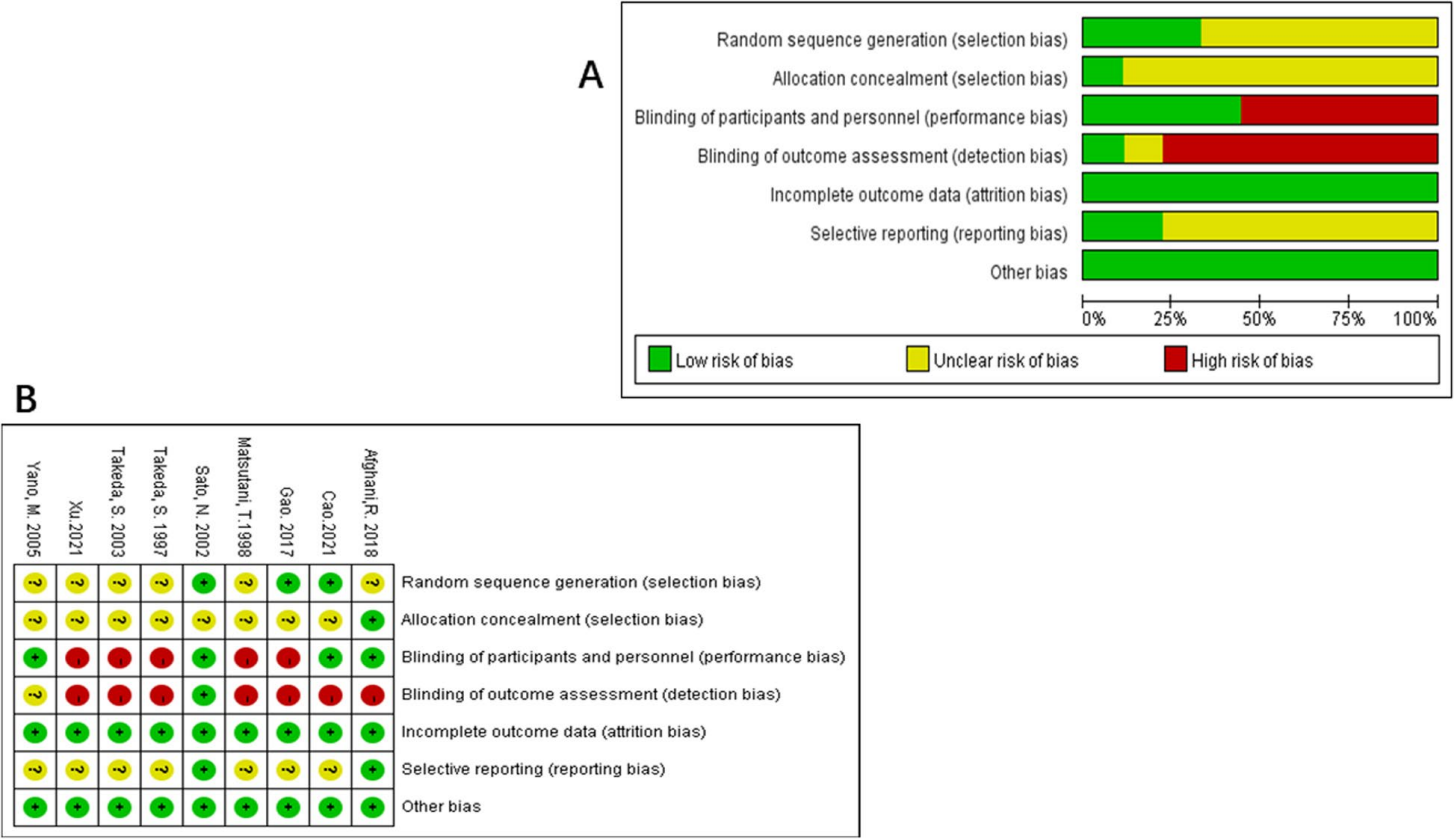
Risk of bias graph (**A**) and summary (**B**)

**Fig. 3:**
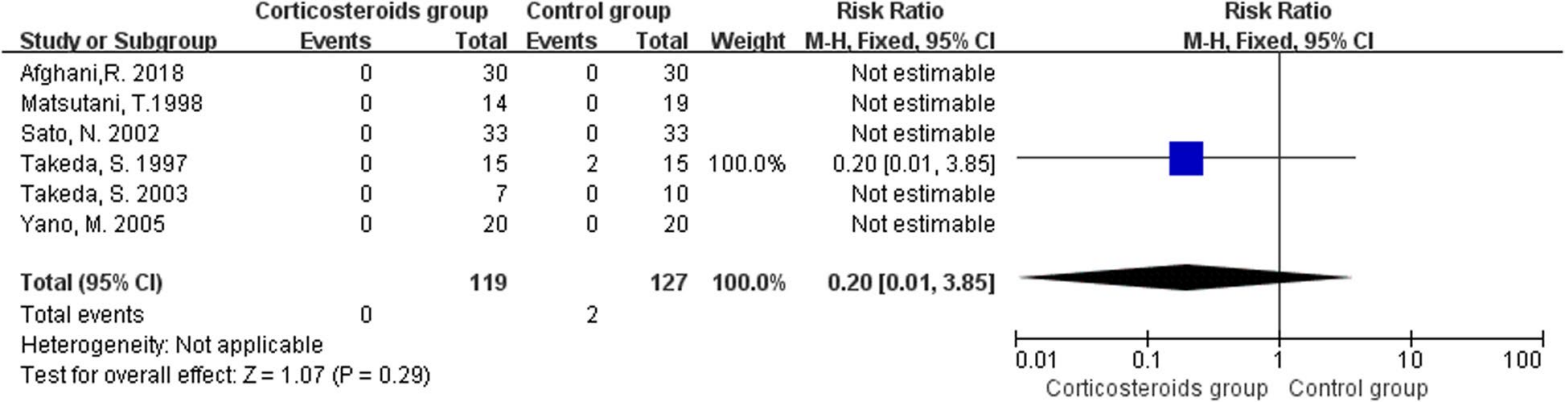
Grading of Recommendations Assessment, Development, and Evaluation (GRADE)

None of the included studies had a low risk of bias in all categories. Risk of bias judgements across studies are summarized in Fig. 2A. The risk of bias for each individual study is summarized in Fig. 2B. GRADE results were presented in Fig. 3.

### Mortality during the hospitalization period

**Fig. 4:**
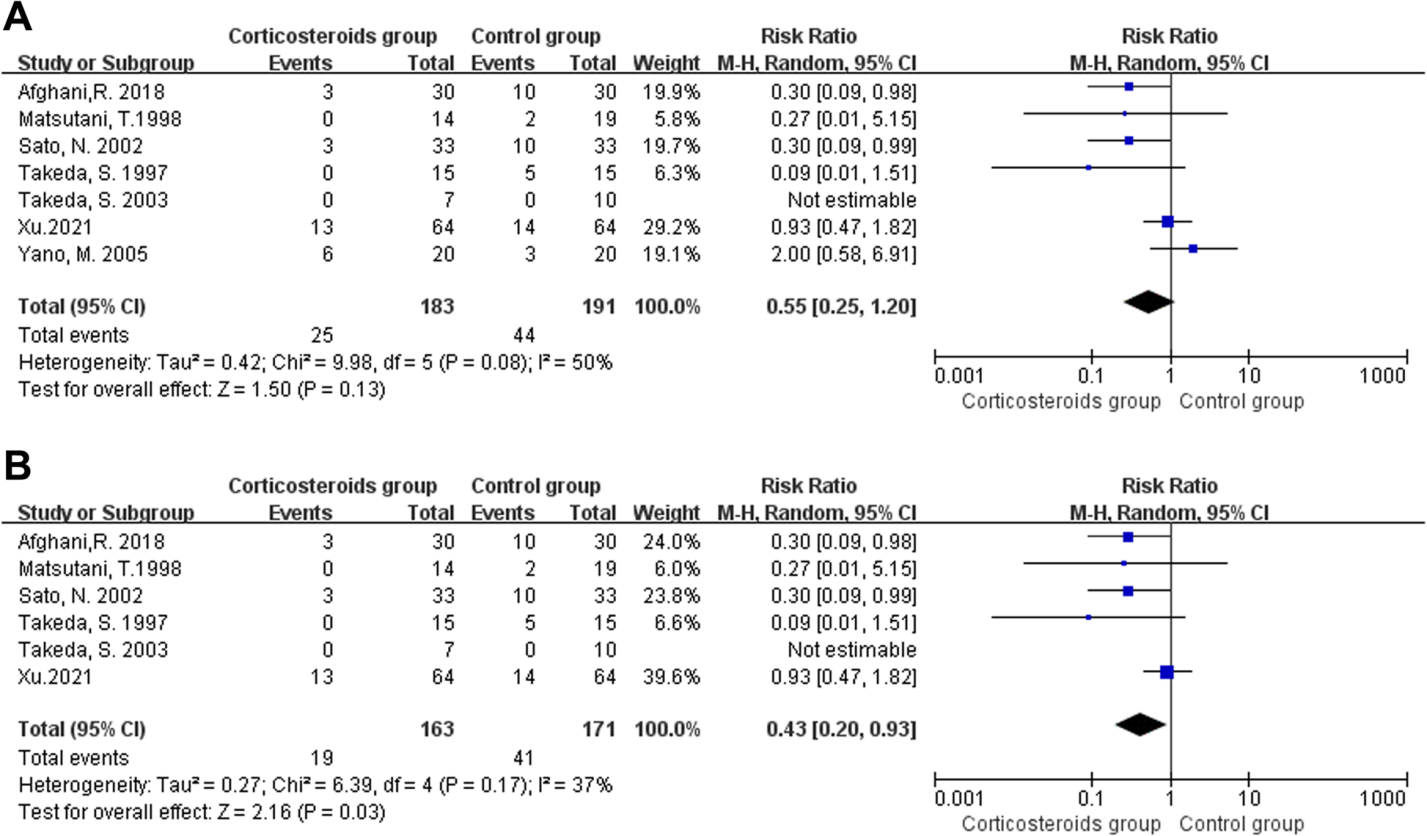
Forest plot for the mortality rate during the hospitalization period

Six studies reported mortality during the hospitalization period in both corticosteroid and control groups. The pooled estimate from the six trials (246 participants) did not confirm the positive effects of corticosteroid administration (RR 0.20, 95% CI 0.01 to 3.85; I^2^ = 0%; Fig. 4) [32, 34, 35, 37, 39, 48]. It should be noted that there were five double-arm-zero-event studies [32, 35, 37, 39, 48], which were automatically discarded from the meta-analysis. Due to the biases in selection, performance and reporting assessments, and wide and imprecise CI, the evidence quality for this outcome was graded as low. All the included trials used methylprednisolone as the treatment drug. The administration dosage used by Afghani et al. [48] was lower than that in the other five trials.

### Length of hospital stay and length of stay in the ICU

Only one article reported differences in the length of hospital stay and the length of stay in the ICU between the two groups [34]. In this trial, the hospital stay (days) in the corticosteroid group was comparable to that in the control group (MD -8.00, 95%CI − 26.29 to 10.29, *P* = 0.39), while the ICU stay (days) in the corticosteroid group was significantly shorter than that in the control group (MD -3.1, 95% CI − 5.43 to − 0.77, *P* = 0.009).

### Pulmonary disorders

**Fig. 5:**
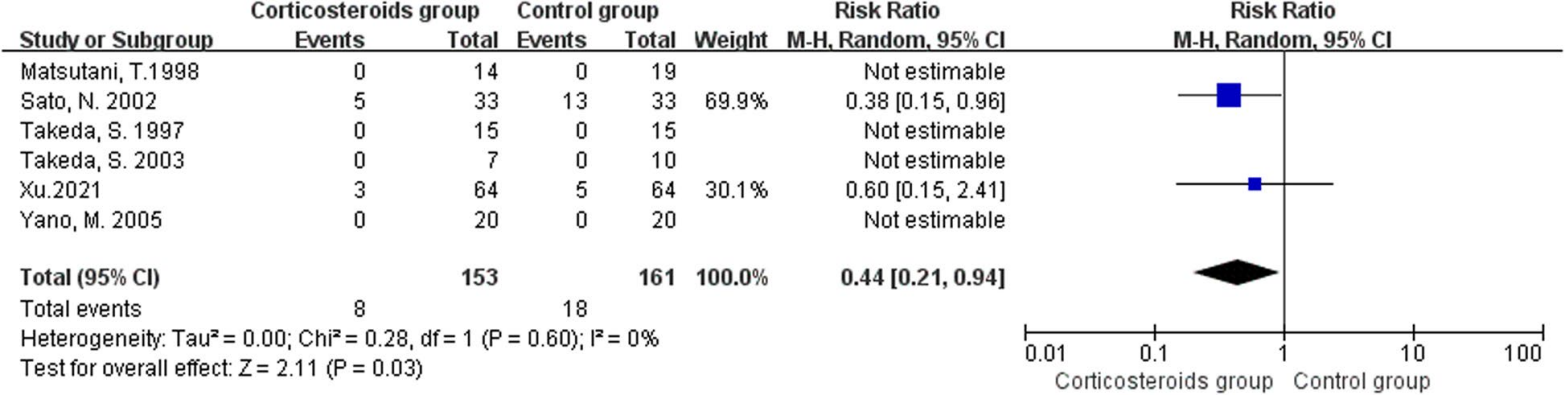
Forest plot for the risk of pulmonary disorder (**A**) and sensitivity analysis (**B**)

Pooled data from seven studies (374 participants) showed methylprednisolone did not affect the rate of pulmonary disorder after esophagectomy (RR 0.55, 95% CI 0.25 to 1.20; I^2^ = 50%,*P* = 0.13; Fig. 5A) [32, 34, 35, 37, 39, 48, 51]. It should be noted that there was one double-arm-zero-event study [35], which was automatically discarded from the meta-analysis. Methylprednisolone was used as the treatment drug in all seven trials. The postoperative pulmonary disorder in the trial by MengKun Cao was identified according to UPSS score, which was continuous value [50]. We could not get the UPSS score of each patient, therefore, this study was not included to calculate the pooled data. Since the value of I^2^ was a little high, we tried to identify the cause of heterogeneity using sensitivity analyses. Afghani et al. [48] used lower dosage, whereas the pulse therapy dosage was used in the other studies. However, a sensitivity analysis with the Afghani study [48] excluded did not change the results. In the trial by Yano et al. [39]. We found the postoperative pulmonary disorders rate in the methylprednisolone group was higher than that in the control group, which was different from the results in the other trials. We further examined the other data of this study. There was an imbalance in age between the methylprednisolone and control groups. The patients in the control group were significantly younger than those in the study group (56 ± 7 vs 64 ± 6 years old; *P* < 0.05). Further sensitivity analysis was conducted with the Yano study [39] excluded, and the results indicated that methylprednisolone had protective effects against pulmonary disorders following esophagectomy (RR 0.43, 95% CI 0.20 to 0.93, *P* = 0.03 I^2^ = 37%; Fig. 5B).

### Cardiovascular disorders

**Fig. 6:**
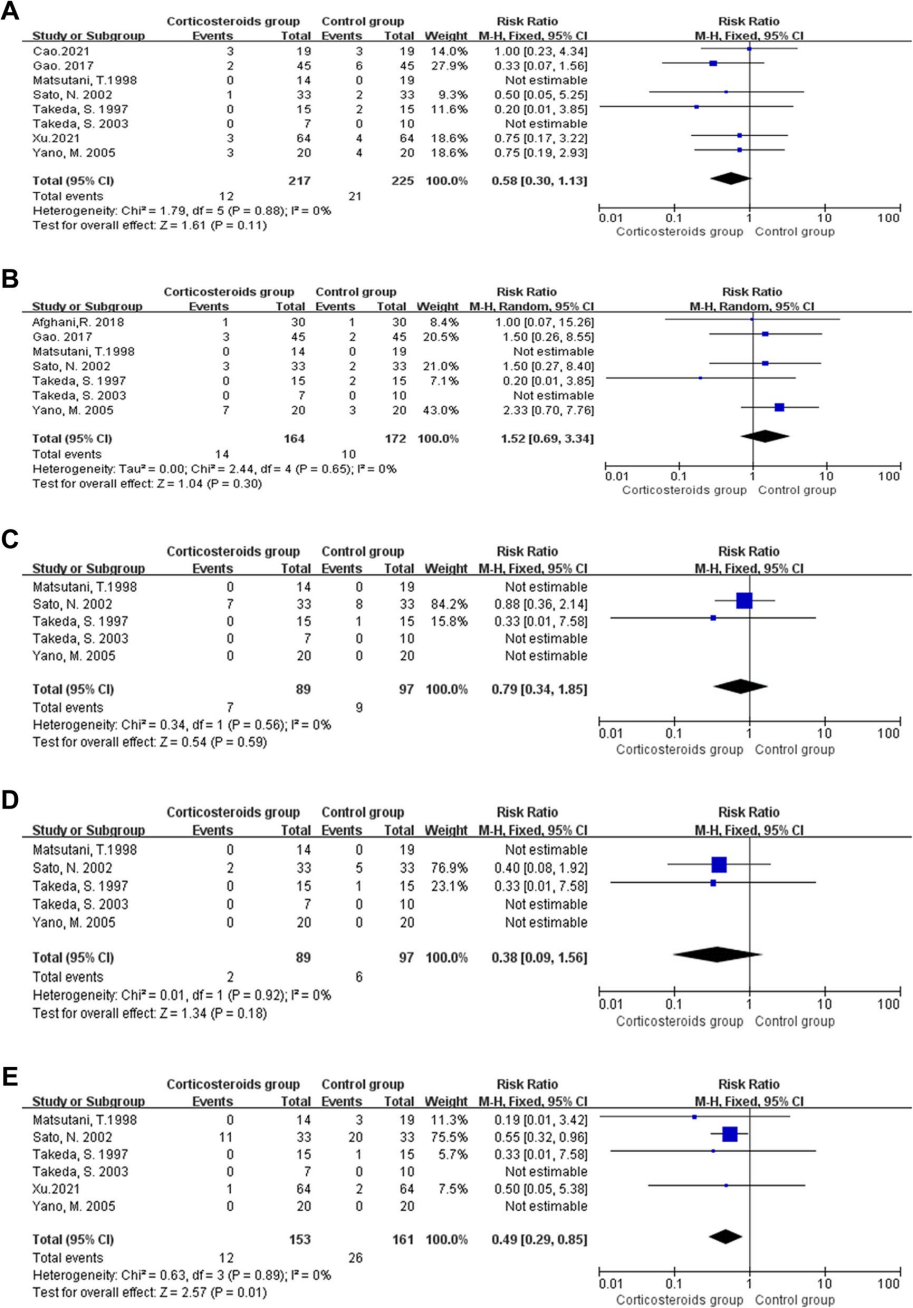
Forest plot for the risk of cardiovascular disorders

Six studies including 314 participants reported cardiovascular disorders, and meta-analysis showed that applying preoperative methylprednisolone decreased the risk of cardiovascular disorders (RR 0.44, 95% CI 0.21 to 0.94, P = 0.03; Fig. 6) [32, 34, 35, 37, 39, 51]. However, four studies [32, 34, 35, 39] had double-arm-zero-events and were automatically discarded from the meta-analysis.

### Anastomotic leakage, general infection complications, renal failure, hepatic failure and other general complications

**Fig. 7:**
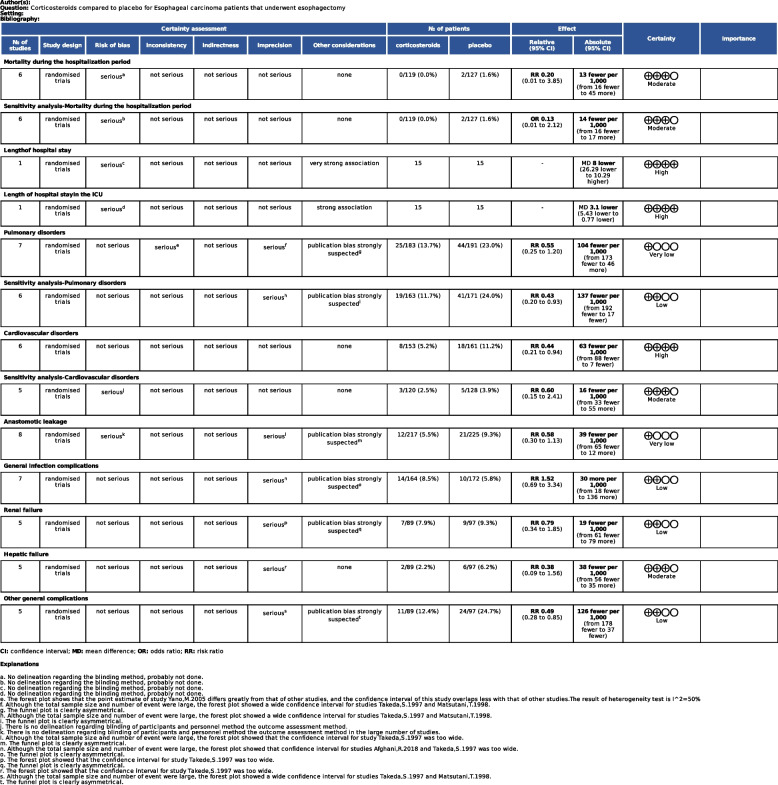
Forest plot for the risk of anastomotic leakage (**A**), general infection complications (**B**), renal failure (**C**), hepatic failure (**D**) and other general complications (**E**)

Meta-analysis of eight studies (442 participants) showed the preoperative methylprednisolone administration had no effects on the incidence of anastomotic leakage following esophagectomy (RR 0.58, 95% CI 0.30 to 1.13; I^2^ = 0%, *P* = 0.11 Fig. 7A) [32, 34, 35, 37, 39, 49–51]. Two of eight studies had double-arm-zero-events and were automatically discarded from the meta-analysis [32, 35]. The majority of the included trials applied methylprednisolone as the treatment drug with the same dosage (at a very high dose level). Gao et al. [49] applied prednisone as a substitute with a dose of 10 mg/kg. According to the administration dosage equivalences reported by Haynes [45], this dose was equal to 8 mg/kg of methylprednisolone.

Seven of nine studies reported general infection complications. Pooled data showed they were not affected by methylprednisolone administration (RR 1.52, 95% CI 0.69 to 3.34; I^2^ = 0%,*P* = 0.30 Fig. 7B) [32, 34, 35, 37, 39, 48, 49]. It should be noted that two studies were automatically discarded from the meta-analysis due to double-arm-zero-events [32, 35]. In the trial by Afghani [48], the methylprednisolone dosage was at a lower level than that in the other trials. The majority of included articles indicated the administration time was before surgery. However, the administration occurred during surgery in the study by Afghani et al. [48] specifically at the end of esophagogastric anastomosis.

Meta analysis of five studies (186 participants) showed no effects of methylprednisolone on renal failure after esophagectomy (RR 0.79, 95% CI 0.34 to 1.85;*P* = 0.59, I^2^ = 0%; Fig. 7C) [32, 34, 35, 37, 39]. The heterogeneity level was low. The methylprednisolone dosage level and administration time points were all the same across the included studies. However, three of five studies reported zero events in both arms and thus were automatically discarded from the meta-analysis [32, 35, 39]. Because the heterogeneity level was low for this outcome, further sensitivity analysis was not conducted. Methylprednisolone was used as the treatment drug at the pulse therapy level in all of the included studies. The administration time points were all before the operation.

There were five studies (186 participants) [32, 34, 35, 37, 39] reported postoperative hepatic failure and other general complications following esophagectomy (314 participants) [32, 34, 35, 37, 39, 51] Meta analyses showed methylprednisolone had no effect on postoperative hepatic failure (RR 0.38, 95% CI 0.09 to 1.56; I^2^ = 0%, *P* = 0.18; Fig. 7D), while may reduce the incidence of other general complications following esophagectomy (RR 0.49, 95% CI 0.29 to 0.85; I^2^ = 0%, *P* = 0.01; Fig. 7E). Three double-arm-zero-events studies [32, 35, 39] were automatically discarded from the meta-analysis of postoperative hepatic failure, while two double-arm-zero-events studies [35, 39] were automatically discarded from the meta-analysis of other general complications. The heterogeneity level of both outcomes was low. The methylprednisolone dosage level and administration time points were same across the majority of included studies.

## Discussion

The reduction of excessive inflammatory cytokine levels by corticosteroids after esophagectomy has been demonstrated by many studies [52, 53]. However, the effects of corticosteroids on postoperative mortality and complications are still on controversial. In this systemic review, all included trials employed methylprednisolone or prednisone, while other types of corticosteroids were not evaluated. The administration routes were all venous and the drug doses were all very high. The study from Afghani gave corticosteroid was during the surgery, and one study from Xu gave the corticosteroid was after surgery. The rest 7 studies all chose the administration time point was before the operation. The sample size of each trial was small and the quality of each included trial was relative low. The enrolled participants were mainly Japanese, Chinese, and Iranians. More than half of included studies were published over 10 years ago. Therefore, conclusions should be made with serious caution for these reasons. In addition, we retrieved a protocal by Magnin et al. [54] that met the literature retrieve requirements. Due to the trial had not been completed, it was not included.

We found three meta-analysis reviews [30, 31, 43] that evaluated the effects of perioperative corticosteroid administration on esophageal cancer patients who underwent esophagectomy. Engelman and colleagues pooled six RCTs and 2 non-RCTs, while the rest three reviews only enrolled RCTs. The following postoperative complications were all focused in each review: respiratory complication, liver dysfunction, anastomotic leak, mortality, sepsis/infection complications, renal dysfunction. The review from Engelman [30] evaluated eight clinical end-points, namely death, respiratory complication, sepsis, liver dysfunction, renal dysfunction, cardiovascular dysfunction, surgical anastomotic leak, and any postoperative organ dysfunction or complication (excluding death), They found that perioperative corticosteroid administration significantly decreased six postoperative complications (any organ dysfunction or complication (death excluded), respiratory complication, sepsis, liver dysfunction, cardiovascular dysfunction, and surgical anastomotic leak). The review from Gao et al. [43] found perioperative corticosteroid administration significantly lowered the incidence of cardiovascular disorders, pulmonary disorders, and failure of any organ. The review from Raimondi et al. [31] showed that the incidence of multiple postoperative complications and respiratory complications was significantly lower in patients who received perioperative corticosteroid administration. The current updated review showed that perioperative corticosteroid administration reduced the risk for cardiovascular disorders and the incidence of general complications. The updated review and two previous meta-analysis reviews from Engelman and Gao [30, 43] suggest that perioperative corticosteroid administration decreased the risk of cardiovascular disorders. The review by Raimondi et al. [31] did not evaluate cardiovascular disorders. All the three previous reviews [30, 31, 43] all suggest perioperative corticosteroid administration reduces the risk for pulmonary disorders/respiratory complications, which is different from the conclusions in the current systemic review. However, we found that postoperative pulmonary disorders may be improved by methylprednisolone after removing a study with potential heterogeneity from age [39]. The discrepancy regarding pulmonary disorders was due to the study [39], in which six patients with pulmonary disorders were identified in the methylprednisolone group with the average age was 63.5 ± 5.6, and three were identified in the control group with the average age was 55.9 ± 6.9. In the meta-analyses by Engelman [30] and Gao [43], the incidence of pulmonary disorders was reversed once enrolled the data from Yano [39], while this study [39] was not included in the review by Raimondi et al. [31] Therefore, a decrease in the incidence of pulmonary disorders in the treatment group was found in the previous three meta-analysis reviews. Since the latest review focusing this theme was published in 2014, the updated review supplied new evidence with enrolling 3 RCTs in time. Besides, this review was registered in Cochrane Upper Gastrointestinal and Pancreatic Diseases Group (196), and conducted under strict criterion following the guideline of Cochrane Handbook for Systematic Reviews of Interventions.

Our study has a few limitations. First, we only included English and Chinese language articles. Second, we have only included randomized controlled trials (RCTs), and other literature types related to the topic, such as cohort studies, protocol, etc., will be excluded. This may lead to the possibility of not covering all relevant literature. Third the current evidence quality was graded as being of low or very low, primarily because of inconsistency, imprecision, and a risk of bias. The imprecision was mainly due to the small number of events and wide 95%CIs. Fourth, the majority of included studies were assessed as having a high risk of bias. Considering the limited applicability of the existing evidence, more RCTs involving different ethnic groups with high quality control and larger sample sizes should be performed in the future.

## Conclusions

The current evidence regarding perioperative corticosteroid administration for esophageal cancer patients with esophagectomy is insufficient to inform clinical practice. We could not ascertain the safety or positive effects of corticosteroid administration on postoperative mortality and complications due to the low and very low quality of the evidence.

### Supplementary Information

**Additional file 1.**

**Additional file 2.**

## Data Availability

The datasets generated and analyzed during the present study are available from the corresponding author upon reasonable request.

## Abbreviations

RCT: randomized controlled trial
RR: risk ratio
CI: confidence interval
MD: mean difference
ICU: intensive care unit
